# Enhancing the differentiation of specific genotypes in *Mycobacterium tuberculosis* population

**DOI:** 10.1038/s41598-019-54393-7

**Published:** 2019-11-29

**Authors:** Shima Hadifar, Mansour Kargarpour Kamakoli, Abolfazl Fateh, Seyed Davar Siadat, Farzam Vaziri

**Affiliations:** 1 Department of Mycobacteriology and Pulmonary Research, Pasteur Institute of Iran, Tehran, Iran; 2 Microbiology Research Center (MRC), Pasteur Institute of Iran, Tehran, Iran

**Keywords:** Microbiology techniques, Microbiology

## Abstract

Today, significant attention is directed towards the global lineages and sublineages of *Mycobacterium tuberculosis* (*Mtb*). NEW-1 (SIT 127) and CAS1-Delhi (SIT 26) strains are recognized as growing and circulating *Mtb* genotypes, especially in Asian countries. It is crucial to develop or enhance *Mtb* genotyping methods for a more accurate and simple differentiation of these families. We used 24-loci mycobacterial interspersed repetitive unit-variable number tandem repeat (MIRU-VNTR) typing for genotyping 217 *Mtb* isolates. To select the optimal MIRU-VNTR loci, we calculated the Hunter-Gaston discriminatory index (HGDI), allelic diversity, and accumulation of percentage differences (APDs) between the strains among different groups of genotypes (NEW-1 and non-NEW-1; CAS1-Delhi and non-CAS). Finally, the minimum spanning tree was constructed for clustering analysis. In the NEW-1 population, loci with APD > 60% were found to have a high discriminatory power. VNTR loci with APD > 50% showed high discrimination power for the CAS population. Our findings suggest that APDs, which are valuable for the selection of VNTR loci sets, may improve the discriminatory power of MIRU-VNTR typing for identification of *Mtb* genotypes in specific regions.

## Introduction

Today, significant attention is directed towards the global lineages of *Mycobacterium tuberculosis* (*Mtb*). Among seven recognized lineages of *Mtb*, lineage 4 is the most widely dispersed, affecting humans around the world^1^. One of the important genotypes in lineage 4 is the NEW-1 family. In addition, NEW-1 (SIT 127) and CAS1-Delhi (SIT 26) strains have been identified as growing and circulating *Mtb* genotypes, especially in Asian countries including Iran, Afghanistan, and Pakistan^2–7^. In these regions, the prevalence of NEW-1 is strongly associated with multidrug resistance (MDR)^7,8^.

The ongoing increase in the circulation of NEW-1 (SIT 127) and CAS1-Delhi strains in West of Asia seems more alarming for the NEW-1 clone population, which is prone to MDR^9^. These reports demonstrate the need for developing or enhancing *Mtb* genotyping methods. Overall, genotyping of *Mtb* strains plays an important role in understanding the dynamics of *Mtb* population, tuberculosis (TB) surveillance, and control programs. The 24-loci mycobacterial interspersed repetitive unit-variable number tandem repeat (MIRU-VNTR) typing is a popular and promising genotyping method for investigating the polymorphisms of *Mtb* DNA minisatellites. Compared to other PCR-based genotyping methods, such as spoligotyping, this approach is less prone to homoplasy and shows a greater discriminatory power^10,11^. Due to the presence of geographic polymorphisms in some VNTR loci, 24-loci VNTR sets may not be able to precisely discriminate local *Mtb* isolates. Therefore, we conducted this study to explore a VNTR loci set for discriminating NEW-1 and CAS1-Delhi (the main sub-lineages of lineages 4 and 3, respectively) from other genotypes by different approaches.

## Results

According to the results of MIRU-VNTR genotyping method, 80 out of 217 *Mtb* isolates were identified as NEW-1 genotype and 95 isolates as CAS1-Delhi genotype. Other isolates belonged to the Beijing genotype. Similar results were also obtained by the spoligotyping method. We aimed to identify appropriate loci with the capacity for precise classification and differentiation of genotypes; therefore, we examined the capacity of the current set of MIRU-VNTR loci.

**Figure 1:**
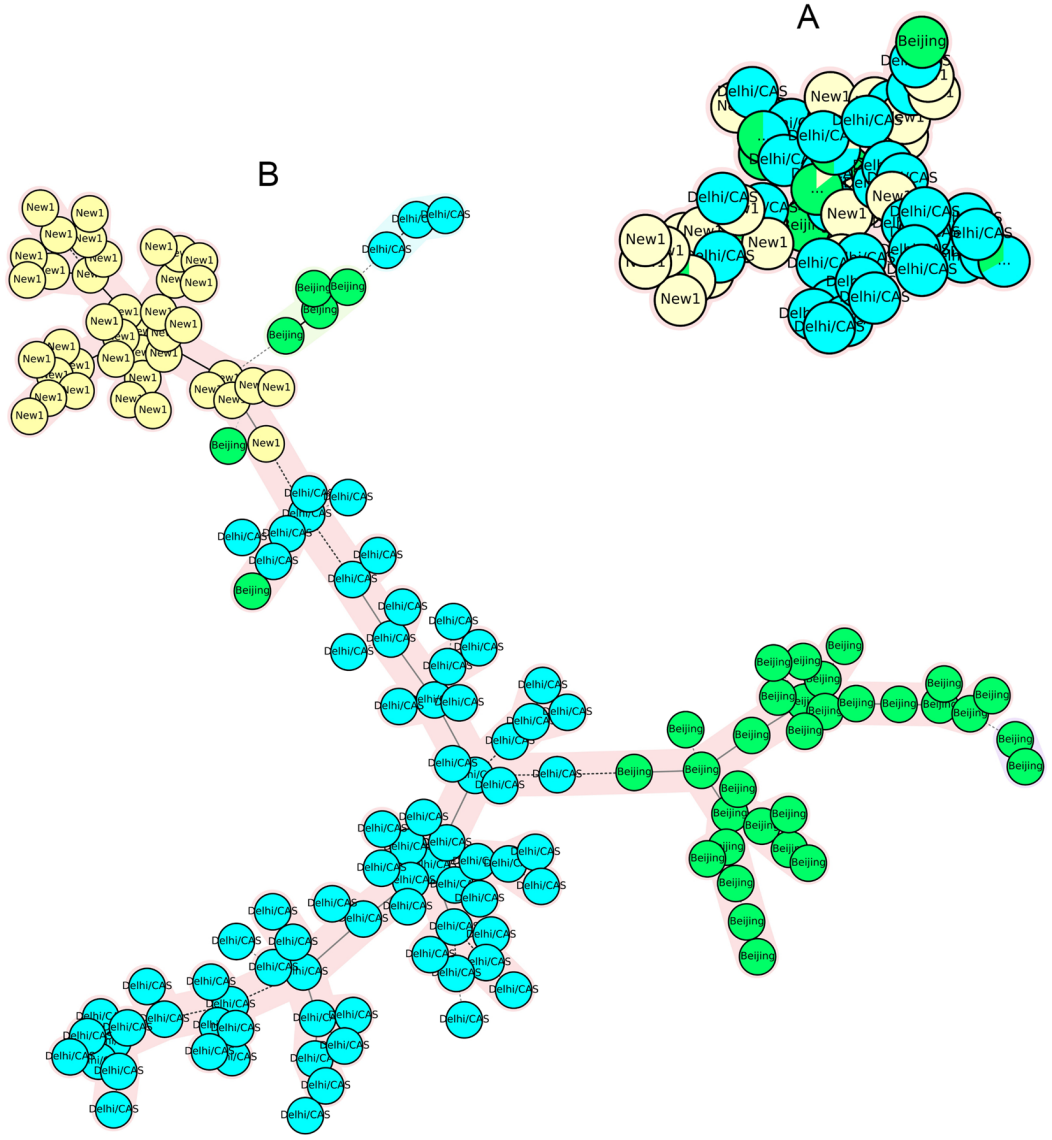
The MSTs of 217 *Mtb* strains based on: (**A**) VNTR loci 4052 (QUB26), 2996 (MIRU26), and 2059 (MIRU20), HGDI > 0.6; (**B**) 960 (MIRU10), 424(Mtub04), 2165 (ETRA), 1644 (MIRU16), 3192 (MIRU31), 2163b (QUB11b), 4348 (MIRU39), 4156 (QUB4156), 577 (ETRC), 3171(Mtub34) and 2401 (Mtub30). NEW-1 and non-NEW-1 strains are shown in different colors.

As shown in Table 1, the discriminatory power of each locus was different between NEW-1, CAS-Delhi, and other genotypes. In the NEW-1 population, VNTRs 4052 (QUB26), 2996 (MIRU26), and 2059 (MIRU20) showed a high degree of discrimination (HGDI > 0.6), whereas 16 VNTR loci were poorly discriminative (HGDI < 0.3). Thirteen VNTR loci showed high discrimination for non-NEW-1 genotypes, three of which, including 4052 (QUB26), 2996 (MIRU26), and 2059 (MIRU20), exhibited a high discriminatory power for the NEW-1 genotype; the remaining loci had low to moderate discriminatory power for this genotype. Eleven VNTR loci showed an HGDI difference ≥0.2 between NEW-1 and non-NEW-1 populations. The MST plot, which was constructed based on highly discriminative VNTR loci and loci with an HGDI difference ≥0.2, showed low potential to classify the genotypes into distinct branches (Fig. 1A,B).

**Table 1:** HGDI of different MIRU–VNTR loci.

MIRU–VNTR locus	Total (n = 217)		CAS-Delhi (n = 95)		Non-CAS (n = 122)		NEW-1 (n = 80)		Non-NEW-1 (n = 137)	
	Diversity Index	CI (95%)	Diversity Index	CI (95%)	Diversity Index	CI (95%)	Diversity Index	CI (95%)	Diversity Index	CI (95%)
2687	0.009	(1.000–0.027)	0	(0.000–0.000)	0.016	(1.000–0.049)	0	(0.000–0.000)	0.015	(1.000–0.043)
2347	0.063	(0.017–0.109)	0.021	(1.000–0.062)	0.096	(0.023–0.169	0.098	(0.007–0.189)	0.043	(1.000–0.092)
154	0.072	(0.024–0.120)	0.102	(0.018–0.187)	0.049	(1.000–0.103)	0.025	(1.000–0.073)	0.099	(0.029–0.169)
580	0.137	(0.077–0.198)	0.156	(0.061–0.251)	0.124	(0.046–0.201)	0.182	(0.075–0.290)	0.111	(0.040–0.182)
3007	0.205	(0.133–0.277)	0.196	(0.090–0.301)	0.212	(0.116–0.308)	0.121	(0.023–0.219)	0.251	(0.157–0.345)
2531	0.2	(0.132–0.269)	0.14	(0.046–0.235)	0.244	(0.152–0.336)	0.074	(1.000–0.154)	0.266	(0.177–0.354)
3690	0.355	(0.277–0.433)	0.357	(0.243–0.470)	0.356	(0.250–0.461)	0.271	(0.146–0.397)	0.4	(0.305–0.496)
3171	0.445	(0.400–0.491)	**0.157**	(0.061–0.254)	**0.504**	(0.498–0.510)	**0.339**	(0.234–0.444)	**0.112**	(0.040–0.183)
2401	0.463	(0.395–0.532)	0.488	(0.385–0.591)	0.447	(0.356–0.537)	**0**	(0.000–0.000)	**0.608**	(0.552–0.663)
2461	0.529	(0.491–0.567)	**0.102**	(0.018–0.187)	**0.488**	(0.416–0.561)	0.049	(1.000–0.116)	0.189	(0.102–0.277)
802	0.481	(0.401–0.561)	0.458	(0.338–0.578)	0.499	(0.394–0.604	0.508	(0.382–0.634)	0.462	(0.362–0.562)
577	0.54	(0.509–0.571)	0.103	(0.017–0.188)	0.126	(0.045–0.208)	**0.097**	(0.008–0.187)	**0.476**	(0.408–0.545)
4156	0.595	(0.536–0.655)	**0.738**	(0.684–0.791)	**0.423**	(0.327–0.519)	**0.283**	(0.164–0.402)	**0.706**	(0.656–0.756)
4348	0.647	(0.605–0.688)	0.678	(0.649–0.706)	0.488	(0.389–0.588)	**0.252**	(0.127–0.377)	**0.693**	(0.673–0.713)
2163b	0.55	(0.474–0.626)	**0.362**	(0.245–0.479)	**0.641**	(0.564–0.718)	**0.286**	(0.164–0.408)	**0.652**	(0.577–0.727)
3192	0.656	(0.608–0.704)	0.728	(0.694–0.761)	0.536	(0.442–0.630)	**0.121**	(0.022–0.219)	**0.741**	(0.720–0.762)
2059	0.617	(0.567–0.667)	0.645	(0.569–0.721)	0.598	(0.532–0.663)	0.611	(0.534–0.687)	0.624	(0.558–0.690)
1644	0.679	(0.643–0.714)	0.487	(0.377–0.597)	0.503	(0.418–0.588	**0.38**	(0.255–0.505)	**0.656**	(0.594–0.719)
2165	0.705	(0.673–0.737)	0.708	(0.646–0.770)	0.637	(0.578–0.697)	**0.395**	(0.272–0.518)	**0.653**	(0.579–0.727)
1955	0.629	(0.589–0.669)	0.598	(0.500–0.695)	0.446	(0.350–0.542)	0.437	(0.310–0.564)	0.623	(0.568–0.678)
2996	0.761	(0.721–0.802)	0.806	(0.768–0.845)	0.632	(0.565–0.700)	0.646	(0.582–0.709)	0.781	(0.737–0.825)
424	0.762	(0.729–0.795)	0.788	(0.745–0.830)	0.617	(0.529–0.705)	**0.273**	(0.146–0.400)	**0.777**	(0.742–0.811)
960	0.79	(0.759–0.821)	**0.772**	(0.732–0.811)	**0.502**	(0.442–0.563)	**0.097**	(0.008–0.187)	**0.8**	(0.772–0.828)
4052	0.837	(0.817–0.856)	0.811	(0.769–0.853)	0.785	(0.741–0.830)	0.684	(0.601–0.768)	0.816	(0.778–0.853)

^*^Bold value represented HGDI value difference ≥0.2.

**Figure 2:**
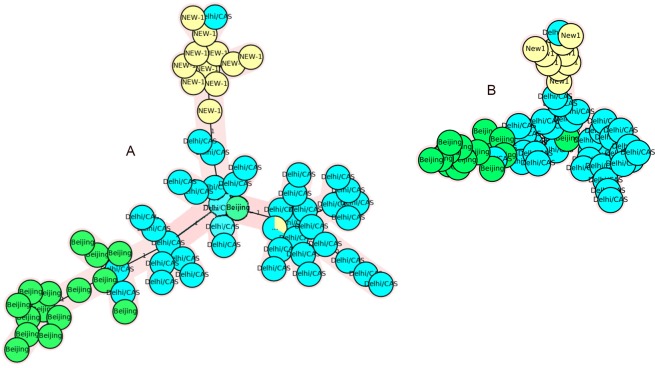
The MSTs of 217 *Mtb* strains based on: (**A**) VNTR loci 4052 (QUB26), 2996 (MIRU26), 424 (Mtub04), 960 (MIRU10), 4156 (QUB4156), 3192 (MIRU31), 2165 (ETRA), 4348 (MIRU39), and 2059 (MIRU20), HGDI > 0.6; (**B**) VNTR loci 960 (MIRU10), 4156 (QUB4156), 2163b(QUB11b), 2461 (ETRB), and 3171 (Mtub34). CAS1-Delhi and non-CAS strains are shown in different colors.

In the CAS-Delhi population, nine VNTR loci, including 4052 (QUB26), 2996 (MIRU26), 424 (Mtub04), 960 (MIRU10), 4156 (QUB4156c), 3192 (MIRU31), 2165 (ETRA), 4348 (MIRU39), and 2059 (MIRU20), showed high discrimination, while the other nine VNTR loci exhibited low discrimination (HGDI < 0.3). VNTR960 (MIRU10), along with 2059 (MIRU20), 3192 (MIRU31), 4348 (MIRU39), and 4156 (QUB4156c), displayed a high discrimination power for the CAS-Delhi genotype and moderate discrimination for non-CAS genotypes. In addition, five loci showed an HGDI difference ≥0.2 between CAS and non-CAS genotypes. The MST, which was constructed based on nine VNTR loci with high discrimination power and five loci with an HGDI difference ≥0.2 in the CAS-Delhi population, failed to classify the genotypes into distinct branches (Fig. 2A,B).

**Figure 3:**
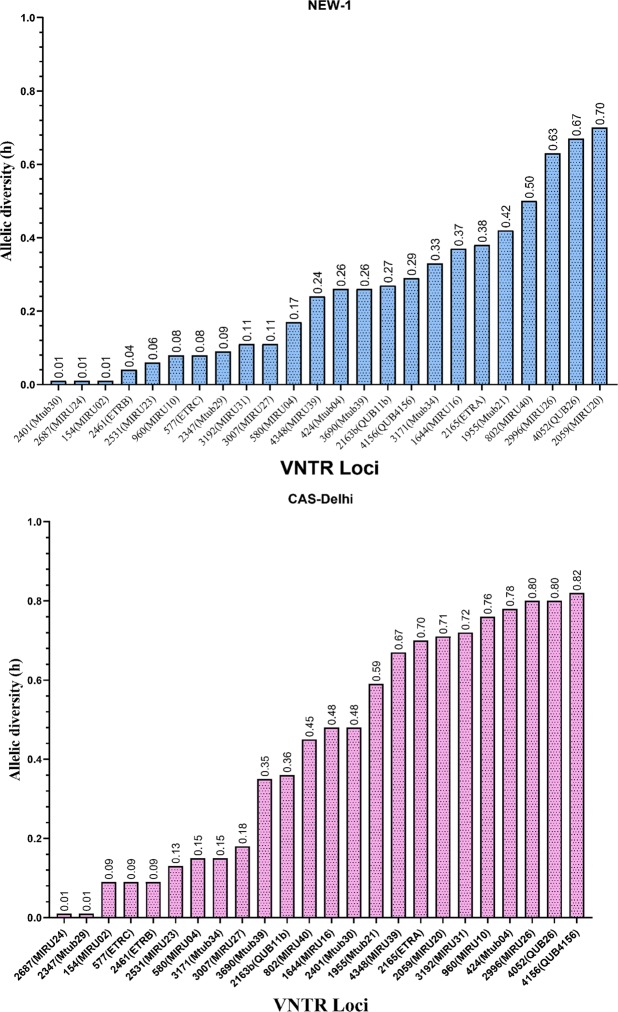
The allelic diversity of each MIRU–VNTR locus.

**Figure 4:**
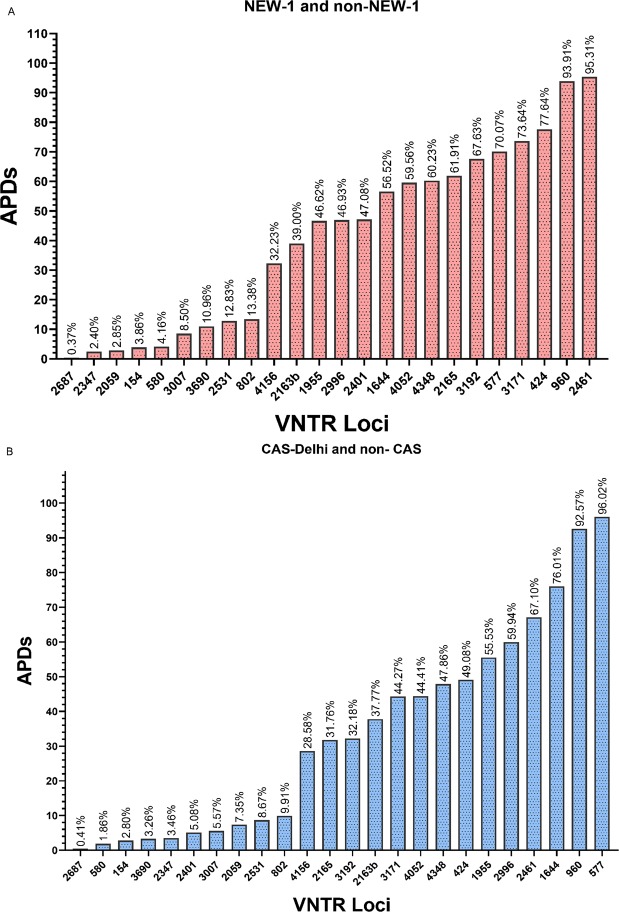
The APDs of 24 VNTR loci between (**A**) NEW-1 and non-NEW-1 strains; and (**B**) CAS-Delhi and non-CAS strains.

The allelic diversity (h) of each VNTR locus is shown in Fig. 3. A different allelic diversity was identified between the NEW-1 and CAS genotypes. The allelic profile of each VNTR locus (copy number of tandem repeats) is shown in Supplementary Figs. S1 and S2. Based on the results, we evaluated the accumulative differences in allelic diversity by calculating APDs to identify the appropriate loci. The APD of each locus related to CAS-Delhi and NEW-1 genotypes is depicted in Fig. 4. In addition, the APD of each locus related to Beijing genotype is illustrated in Supplementary Fig. S3.

**Figure 5:**
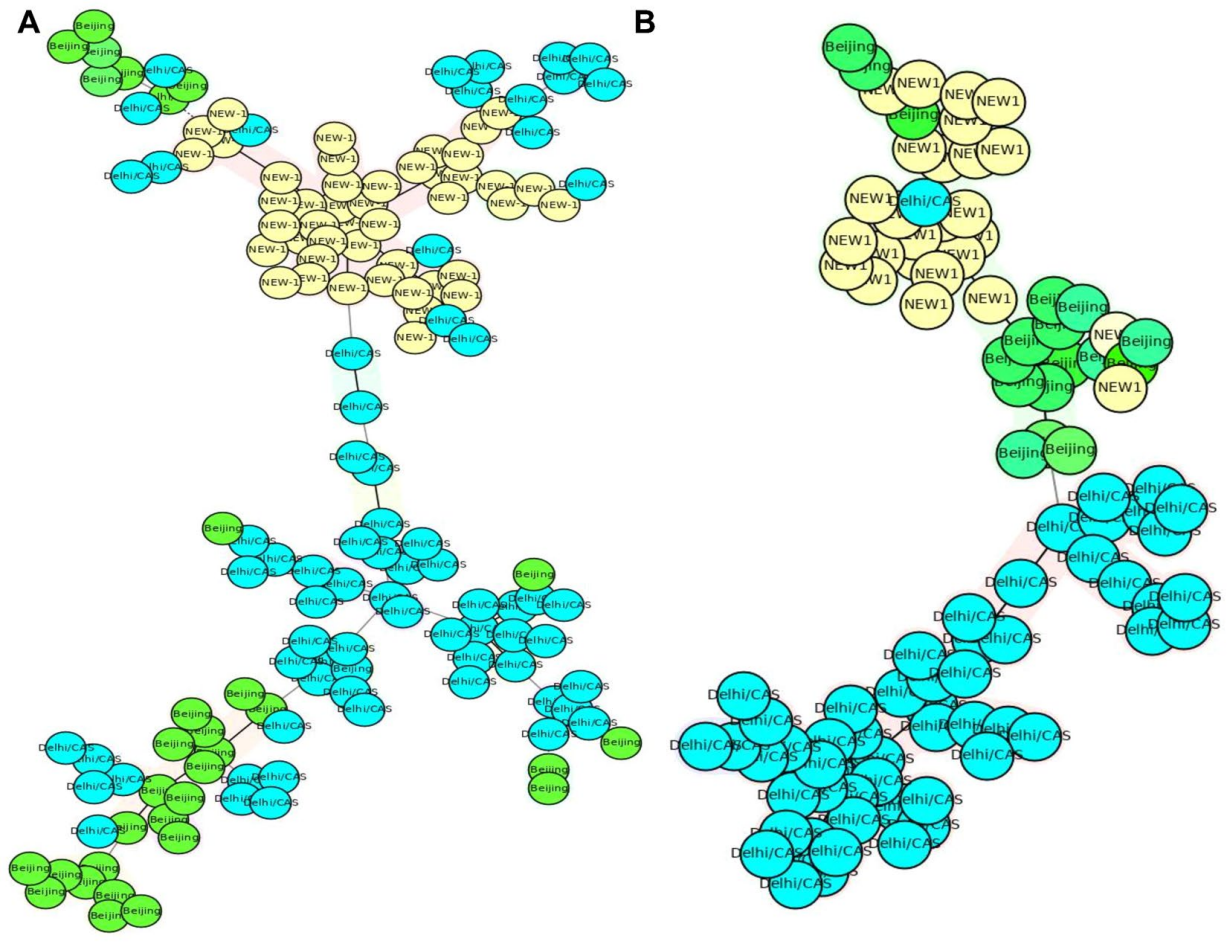
The MSTs of 217 *Mtb* strains based on: (**A**) VNTR 577 (ETRC), 960 (MIRU10), 1644 (MIRU16), 2461 (ETRB), 2996 (MIRU26), and 1955 (Mtub21); (**B**) VNTR 2531 (MIRU23), 802 (MIRU40), 4156 (QUB4156), 2165 (ETRA), 3192 (MIRU31), 2163b (QUB11b), 3171 (Mtub34), 4052 (QUB26), and 4348 (MIRU39). CAS1-Delhi and non-CAS strains are shown in different colors.

**Figure 6:**
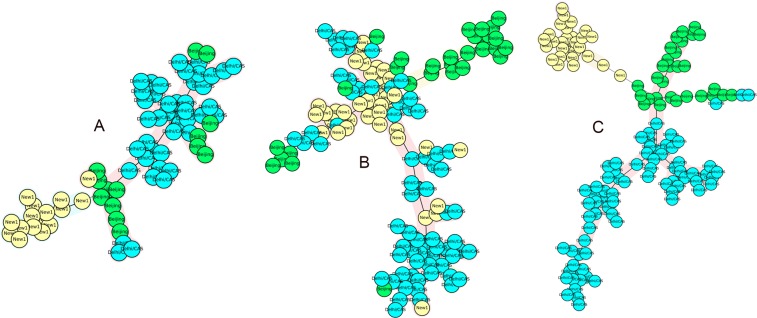
The MSTs of 217 *Mtb* strains based on: (**A**)VNTR loci 2461 (ETRB), 960 (MIRU10), 424 (Mtub04), 3171 (Mtub34), and 577 (ETRC); (**B**) VNTR 3007 (MIRU27), 3690 (Mtub39), 2531 (MIRU23), 802 (MIRU40), 4156 (QUB4156), 2163b (QUB11b), 1955 (Mtub21), 2996 (MIRU26), and 2401 (Mtub30); (**C**) VNTR loci 2461 (ETRB), 960 (MIRU10), 424 (Mtub04), 3171 (Mtub34), 577 (ETRC), 3192 (MIRU31), 2165 (ETRA), and 4348 (MIRU39). NEW-1 and non-NEW-1 strains are shown in different colors.

The results of MST analysis, based on the APD of each locus, demonstrated that VNTR 577 (ETRC), 960 (MIRU10), 1644 (MIRU16), 2461 (ETRB), 2996 (MIRU26), and 1955 (Mtub21) could classify CAS-Delhi and non-CAS genotypes into two main populations. It was found that 93 out of 95 (97.89%) CAS isolates were in the CAS-Delhi population branch. The APDs of the applied VNTR loci were above 50%. MST, based on APDs ranging from 8% to 47%, was also constructed (Fig. 5A,B). The results showed that the VNTR loci set had a low capacity for classification of CAS-Delhi and non-CAS genotypes into distinct branches, while it showed promising capacity for clustering the NEW-1 genotype. This potential is attributed to the presence of five VNTR loci with high APD values for clustering the NEW-1 population. We found loci with APDs > 50% and high discrimination power for the CAS population. In NEW-1 and non-NEW-1 genotypes, VNTR loci, including 2461 (ETRB), 960 (MIRU10), 424 (Mtub04), 3171 (Mtub34), and 577 (ETRC), could classify these isolates into two distinct groups. Out of 80 NEW-1 strains, 79 (98.75%) were found in a distinct branch. In this classification, APDs of VNTR loci were above 70%. The same result was obtained using eight loci with APDs ranging from 60% to 95%. The MST, based on the other VNTR sets with an ascending order in terms of APD, showed different results and low capacity for clustering different genotypes in a specific branch (Fig. 6A–C). In the NEW-1 population, loci with APD > 60% were found to have a high discriminatory power.

## Discussion

The allelic diversity and discriminatory power of VNTR loci in 15/24-loci MIRU-VNTR typing have been evaluated in different studies. The results of these studies showed that the discriminatory power may be affected by the geographical distribution of isolates and strain polymorphisms in specific regions. Some studies have suggested several optimized VNTR sets, mainly VNTRs with high discriminatory powers, for improving the standard *Mtb* typing method and identifying certain genotypes^3,12–16^. In our study, the prominent genotypes belonged to CAS1-Delhi (SIT 26) and NEW-1 (SIT 127) families. The dispersal of NEW-1/SIT127 shows that the family initially emerged in Iran and then spread to other geographical regions^9,17^.

A set of eight optimized VNTR loci, including 4052 (QUB26), 2163B (QUB11b), 802 (MIRU40), 4165 (QUB4156), 2165 (ETRA), 960 (MIRU10), 1644 (MIRU16), and 2996 (MIRU26), with high HGDI values, was identified by Asante-Poku *et al*. for identification of lineages 4 and 5 (*M. africanum* West Africa-1) in Ghana^18^. In addition, Ali *et al*. reported that MIRU loci 10, 16, 26, 27, 31, 39, and 40 showed high discriminatory power for separating predominant CAS1 strains in Pakistan^15^. In a study which conducted in Madagascar, VNTR loci 960 (MIRU10), 4156 (Qub4156), 1644 (MIRU16), 802 (MIRU40), 577 (ETRC), 3690 (Mtub39), 4052 (Qub26), 2163b (Qub11b), 3192(MIRU31) and 424 (Mtub04) have been reported as a set of ten optimized VNTR loci for discrimination of CAS sub-lineage based on HGDI value. In addition, QUB26, Mtub39, QUB4156, ETR-A, ETR-D, ETR-E, QUB11b, Mtub21, and MIRU40; QUB11b, MIRU10, QUB4156, and MIRU40; QUB26, MIRU40,and QUB11b; and Mtub39, MIRU16, QUB11b, MIRU26, ETR-C, ETR-A Qub26, MIRU40, Mtub21, MIRU10, Mtub04, QUB4156 and Mtub30 have been proposed as the set of MIRU-VNTR loci combination for discrimination of EIA, Haarlem, LAM and T genotypes, resectively^19^. In this study, we investigated the discriminatory power of different numbers and sets of VNTR loci to identify a combination of loci for discriminating CAS and NEW-1 from other strains. Besides 4052 (QUB26) and 2996 (MIRU26), Seven VNTR loci, namely 424 (Mtub04), 960 (MIRU10), 4156 (QUB4156), 3192 (MIRU31), 2165 (ETRA), 4348 (MIRU39), and 2059 (MIRU20), showed high discriminatory power in an ascending order for the separation of CAS-Delhi. On the other hand, the MST analysis based on this set showed low capacity for separation of CAS population in a distinct branch. A similar result was found in the MST analysis of the NEW-1 population.

For improving the loci set selection, we examined APDs, i.e., the relative difference between strains with the same repeat number at a specific VNTR locus, between CAS and non-CAS, as well as NEW-1 and non-NEW-1 strains. We found that a six-loci combination with APDs ranging from 55.53% to 96% had significant power to discriminate the CAS family, compared to another set with APDs of less than 50%. In addition, we found two VNTR loci, namely 4052 (QUB26) and 2996 (MIRU26), with extremely high discrimination values (HGDI > 0.8) and high allelic diversity (h) for CAS classification. The VNTR locus 2996 (MIRU26) showed an APD above 55%. Considering their variations, these two loci would be excluded. Nevertheless, the locus APD showed that this locus might contribute to the definition of an effective locus set for determination of CAS strains.

In NEW-1 strains, an effective classification was obtained using a five-loci combination with APDs above 70% and an eight-loci combination with APDs above 60%. In a previous study, the ETR-B (VNTR 2461) locus was proposed as a preliminary marker for identifying the NEW-1 genotypes^17^. The present results showed that this VNTR locus had low allelic diversity and discriminatory power, while it showed the highest APD (95.31%) and was a member of VNTR locus set, which efficiently discriminated NEW-1 (SIT127) strains from non-NEW-1 strains. Moreover, these combination set in both genotypic groups have not been reported for discrimination of other *M.tb* sub-lineages. In a similar study, Pan *et al*. reported that use of APDs facilitates the identification of effective VNTR loci to discriminate the Beijing genotype as the most common family in China^20^. In the current study, we also used APD for the identification of effective VNTR loci to discriminate of Beijing strains from non-Beijing strains and we found that MIRU10, QUB11b, Mtub30, ETRA, ETRC, and MIRU31 had the APD value above 50% while Pan’s study^20^ showed QUB18, MIRU31, Mtub30, Mtub21, MIRU10, and MIRU39 were effective VNTR loci set for discrimination of the Beijing genotypes. The specific geographical setting, the particular circulating sub-lineage and the different sample size may explain this difference in the proposed VNTR loci combination.

Our findings suggest that APDs, which are valuable in the selection of VNTR loci sets, may improve the discriminatory power of MIRU-VNTR typing for identification of *Mtb* genotypes (NEW-1 and CAS1-Delhi) in specific regions. However, further investigations with combined SNPs and VNTR typing schemes can improve the identification and classification of these monomorphic bacterial pathogens.

## Methods

### Isolate collection

We randomly collected 217 *Mtb* isolates from individuals with definite pulmonary TB infection from January 2015 to January 2017. The sample included 84 isolates, which had been identified in our previous study^2^. The Ethics Committee of Pasteur Institute of Iran approved this study. All experiments were performed in accordance with guidelines approved by Pasteur Institute International networks. Ethical reviews and informed consent approval were granted by the Ethical Committee of the Pasteur Institute of Iran. Informed consent was obtained from all patients in this study. The results of this research did not influence TB patients’ treatment.

### Twenty-four-loci MIRU-VNTR typing

The genomic DNA of all inactivated *Mtb* isolates was extracted using a PROBA-NK DNA extraction kit (DNA Technology Company, Moscow, Russia) according to the manufacturer’s instructions and analyzed, based on the 24-loci MIRU-VNTR genotyping method described by Supply *et al*.^21^. We calculated the allelic diversity (h)^22^ and discriminatory power of each VNTR locus, using the Hunter-Gaston discriminatory index (HGDI), as a measure of variation in the number of repeats^23^ for all *Mtb* isolates. We considered *h* > 0.6 as highly discriminative, 0.3 ≤ *h* ≤ 0.6 as moderately discriminative, and *h* < 0.3 as poorly discriminative^24^. The HGDI varies between zero and one and represents the discriminatory power for VNTR loci in combination. Besides, accumulation of percentage differences (APDs) was calculated in each VNTR locus, as described previously^20^.

### Spoligotyping

Spoligotyping was performed using a Spoligotyping commercial kit (Mapmygenome Genomics Company, India), following the protocol described by Kamerbeek *et al*.^25^.

### Data analysis and statistical analysis

The MIRU-VNTR results were analyzed in MIRU-VNTRplus (http://www.miru vntrplus.org/MIRU/index.faces)^26^, TBminer databases (http://infodemo.lirmm.fr/tbminer/about.php)^27^ and StackTB (https://stacktb.cs.sfu.ca/)^28^. Based on each VNTR loci set, a Minimum Spanning Tree (MST) was constructed using MIRU-VNTRplus database to determine genetic variations and classify the isolates. SITVIT-WEB (http://www.pasteur-guadeloupe.fr:8081/SITVIT_ONLINE/index.jsp)^29^ and TBminer databases were also used to analyze the spoligotyping data.

Index of Diversity and confidence intervals were calculated by using the Web tools: (http://www.comparingpartitions.info/index.php) and (http://www.hpa-bioinfotools.org.uk/cgi-bin/DICI/DICI.pl).

### Ethics approval and consent to participate

Ethical reviews and informed written consent were granted by the Ethical Committee of the Pasteur Institute of Iran (Under the Ethics code of 927).

## Supplementary information

Supplementary Figures

## Data Availability

The datasets used and/or analyzed during the current study could become available through the corresponding author on reasonable request.
