# Supplementary material for: Enhancing the differentiation of specific genotypes in Mycobacterium tuberculosis population
Source: Sci Rep. 2019 Nov 29;9:17946. doi: 10.1038/s41598-019-54393-7 (PMC6884525; doi:10.1038/s41598-019-54393-7)
Supplement: Supplementary file 1 — Supplementary Figures [file 41598_2019_54393_MOESM1_ESM.pdf]

Enhancing the differentiation of specific genotypes in *Mycobacterium tuberculosis* population

Shima Hadifar [#], Mansour Kargarpour Kamakoli [#], Abolfazl Fateh, Seyed Davar Siadat, Farzam Vaziri *

[#]These authors contributed equally to this work.

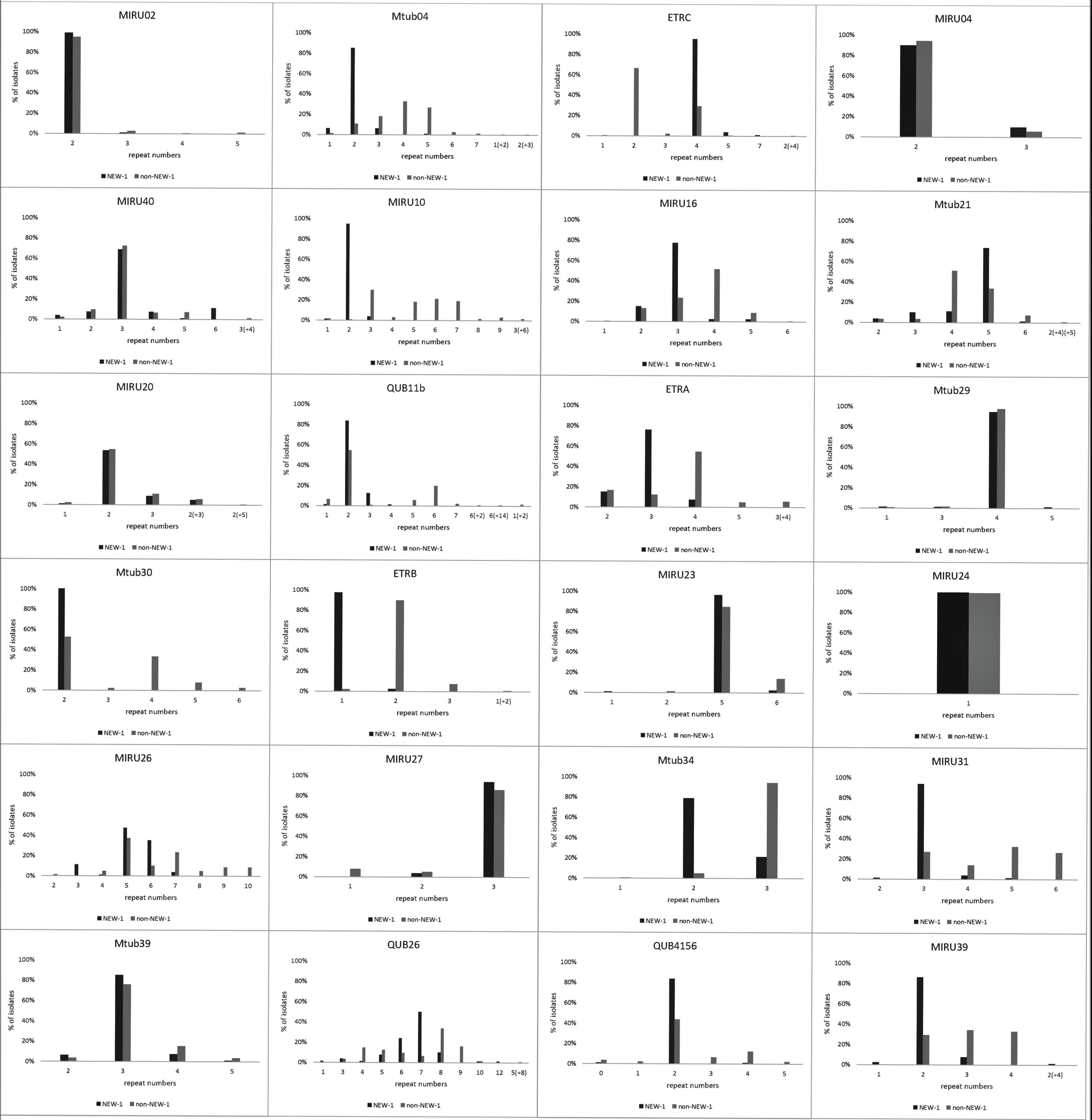

Supplementary Figure S1. Allelic profile of the MIRU-VNTR loci between NEW-1 (black) and non-NEW-1 (gray) strains.

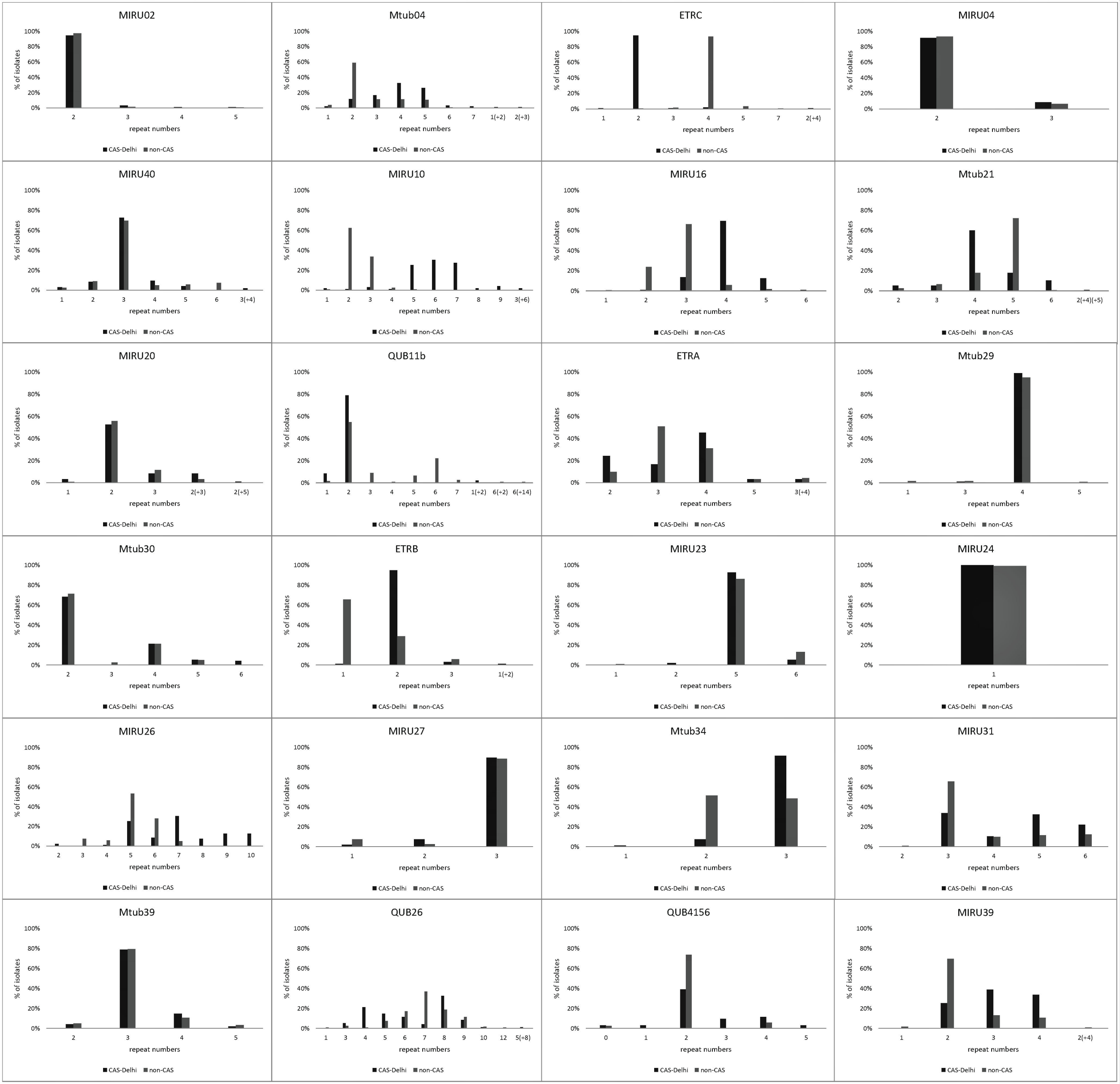

Supplementary Figure S2. Allelic profile of the MIRU-VNTR loci between CAS-Delhi (black) and non-CAS (gray) strains.

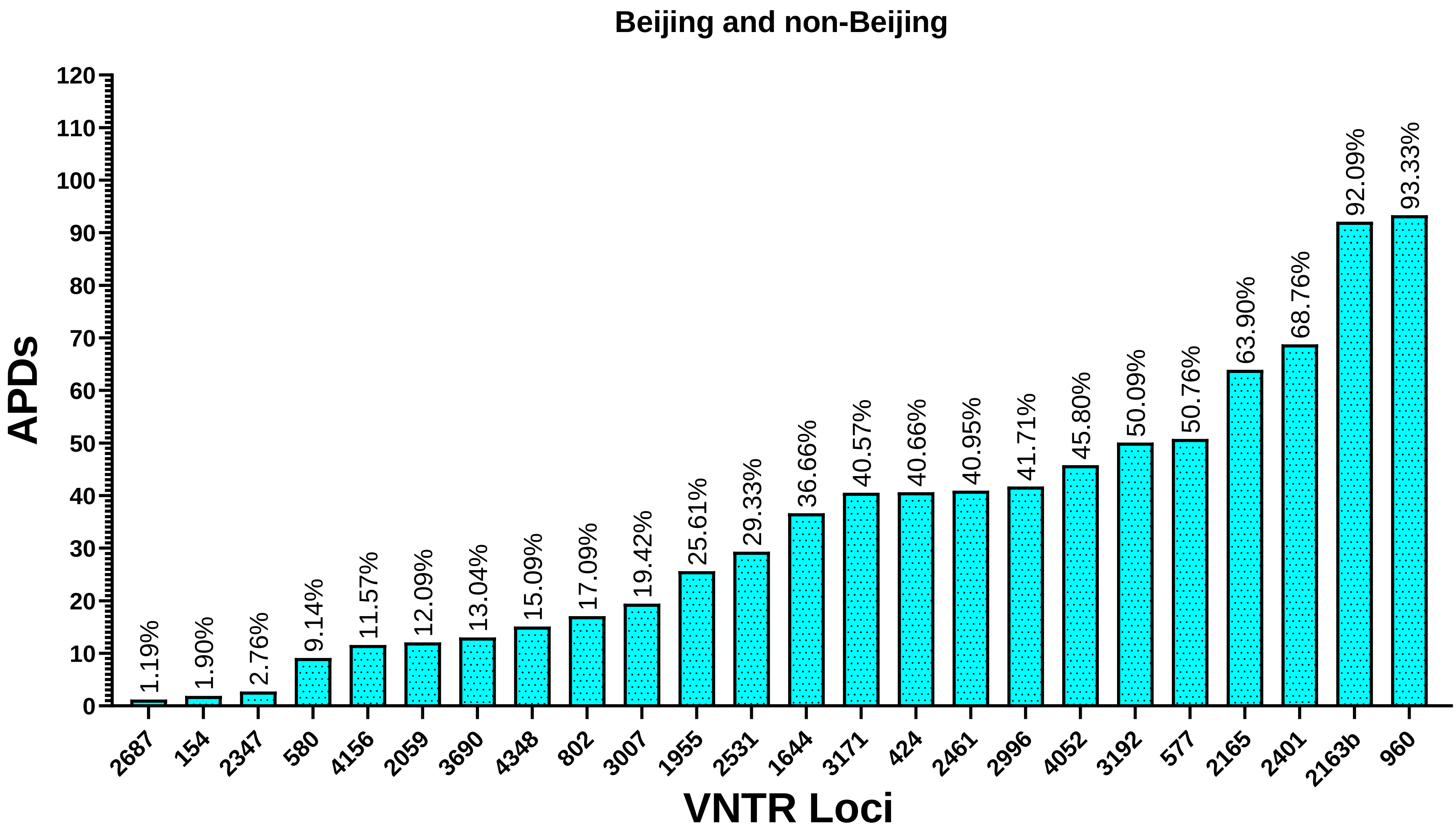

Supplementary Figures S3. The APDs of 24 VNTR loci between Beijing and non-Beijing strains.
